# Iranian primary health care network: challenges and ways forward

**DOI:** 10.1017/S1463423622000354

**Published:** 2023-01-09

**Authors:** Leila Doshmangir, Ahmad Shirjang, Abraham Assan, Vladimir Sergeevich Gordeev

**Affiliations:** 1 Tabriz Health Services Management Research Center, School of Management& Medical Informatics, Tabriz University of Medical Sciences, Tabriz, Iran; 2 Social Determinants of Health Research Center, Tabriz University of Medical Sciences, Tabriz, Iran; 3 National Center for Health Insurance Research, Tehran, Iran; 4 Global Policy and Advocacy Network (GLOPLAN), Accra, Ghana; 5 Institute of Population Health Sciences, Queen Mary University of London, London, UK; 6 Department of Infectious Disease Epidemiology, London School of Hygiene & Tropical Medicine, London, UK

**Keywords:** community health, family physician, family practice, health policy and system research, Iran, primary health care

## Abstract

**Aim:**

This study aimed to explore the current challenges of Iran’s Iranian Primary Health Care (PHC) network and possible ways forward.

**Background:**

PHC network was established in 1985. It remains a core instrument of health care delivery. However, it faces several challenges that can threaten its effective functioning.

**Methods:**

We conducted face-to-face semi-structured interviews with 26 key stakeholders. We used the deductive content analysis approach. World Health Organization’s health system framework guided our analyses. Data were analysed using MAXQDA software. To enhance data triangulation, we reviewed PHC national related plans, bylaws, and national and international published reports.

**Findings:**

PHC network experiences financial challenges and fails to respond fully to the emerging population’s needs due to unfair distribution of resources and a lack of community health workers for PHC and a sustainable financing model for PHC. Furthermore, the insurance package is not well integrated into the PHC network system. Policy interests and resource commitments for innovative, preventive, and health promotion initiatives are lacking. Innovative, preventive, and health promotion initiatives should become the highest priority for policymakers. Well-trained community health professionals, active community participation, private sector engagements and active involvement of non-government organisations are fundamental for a well-functioning PHC network in Iran, especially to foster the delivery of evidence-based initiatives.

## Background

Based on the Alma-Ata Declaration of 1978, health is a public right. It is widely agreed that one of the core functions of government is to ensure adequate provision of such a good (Park, 2005). As highlighted in Article 25 of the Universal Declaration on Human Rights, primary health care (PHC) has been proven to be a highly effective and efficient instrument rooted in a commitment to achieving social justice and equity and the recognition of the fundamental right to the highest attainable standard of health (Rawaf *et al*., 2008; Van Lerberghe, 2008). Further, the Health Promotion Conference of the World Health Organization (WHO) recognised “Health for All” as a framework for developing a future health policy by affirming the importance of public health care in sustainable health development (Van Lerberghe, 2008). Following the Declaration, most countries created PHC networks to optimise their health services delivery.

The Iranian PHC network was designed between 1974 and 1985 to foster context-specific and socially adequate basic health care (Shadpour, 2000). The creation of the PHC network yielded several health gains, including increased vaccination coverage, reduced mother and children’s mortality, and better control of infectious diseases (Javanparast *et al*., 2011b; Malekafzali, 2014). However, some challenges remain, including a centralised decision-making process (Moghadam *et al*., 2012; Lankarani *et al.*, 2013), parallel service delivery (Moghadam *et al*., 2012), lack of knowledge regarding the health package’s content, a discrepancy between existing and required skillset to provide these services among the staff (Shadpur, 2006; Moghadam *et al*., 2012), and a lack of timely health needs assessment that would capture emerging and changing population needs (Sadrizadeh, 2001; Lankarani *et al.*, 2013). In the current Iranian PHC system, the public sector provides a considerable part of PHC services. The private sector has also been involved in PHC service provision (Mehrdad, 2009).

**Figure 1.**
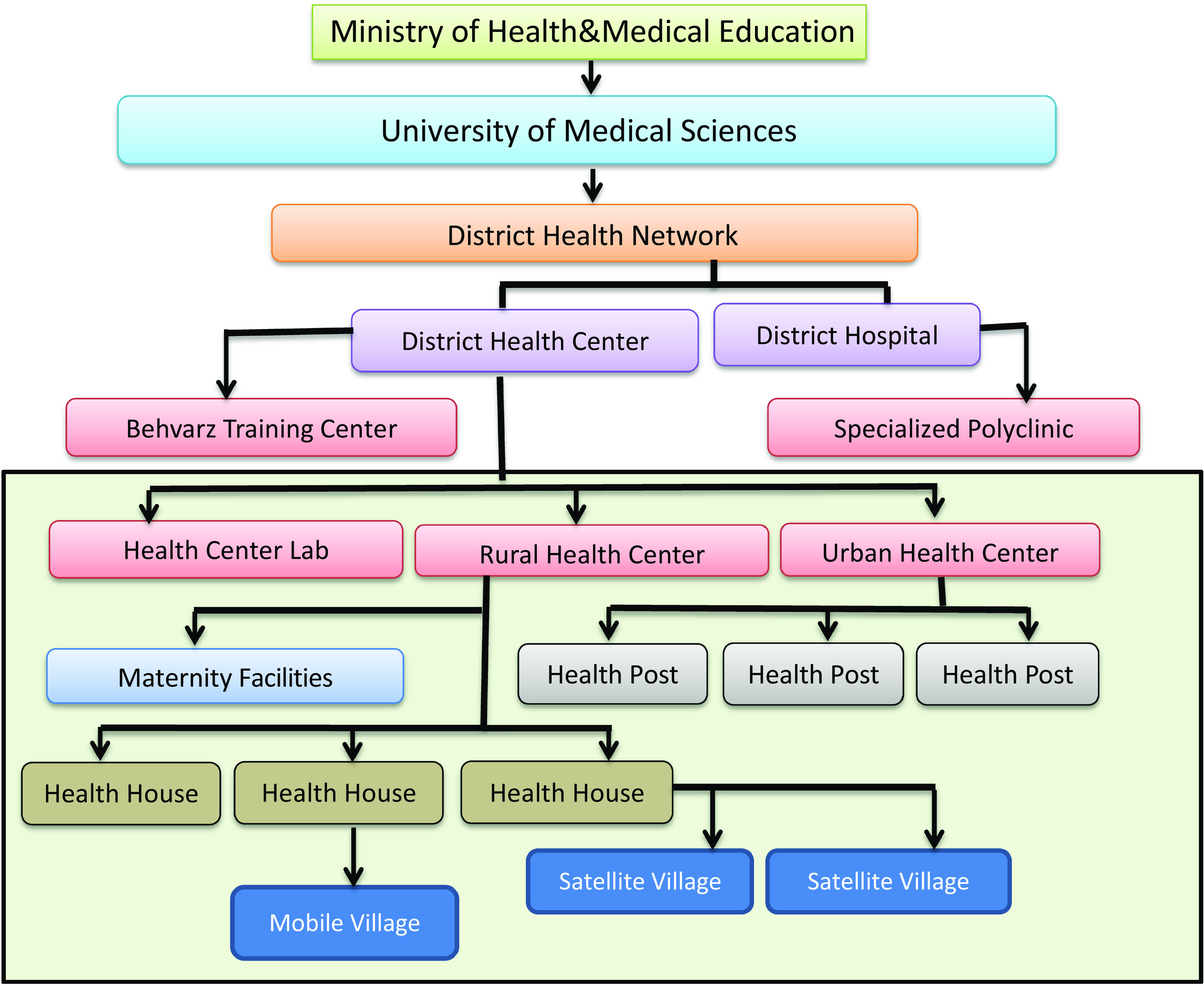
Structure of PHC Network in Iran.

In the first level of the PHC system, a trained community health worker (Behvarz in Persian) provides health services. Each village (sometimes several villages) has a health house run by a Behvarz. Behvarzes are the most fundamental element of the health system, and well familiar with the culture and traditions and have an in-depth understanding of social determinants of health. Behvarzes were trained to meet the basic healthcare needs and improve the rural community’s health (Doshmangir *et al*., 2019). Their main tasks include identifying the rural population, caring for pregnant mothers, children, the elderly, and the middle-aged, preventing and caring for communicable and non-communicable diseases, nutrition and providing services like vaccination services to the target population, environmental and occupational health, cooperation with a family physician, first aid, simple symptomatic treatments, school health and oral health. In large villages, there are also rural health centres in addition to a health house. Health centres are run by general practitioners, have pharmacies and laboratories, and provide essential medical services, including medicine, dentistry, midwifery, screening, care for diabetes and hypertension patients, medical tests, and nutritional and psychological counselling. They are staffed by a qualified physician and a team of up to 10 health care providers providing some more sophisticated primary care. Health posts and health centres in urban areas offer services similar to those of health houses and rural health centres. The region’s health centres manage the PHC network under medical universities’ supervision (Malekafzali, 2014; Tabrizi *et al.*, 2017) (see Figure 1).

The payment mechanism to providers in PHC varies depending on the type of service, the level of service provided, the type of ownership of the institutions, and the type of insurance. The payment system for family physicians who offer prevention services is the payroll system. The payment system for services provided in offices and clinics is based on a fee-for-service reimbursement system. However, service providers can receive salaries besides fee-for-service depending on the type of ownership. In the inpatient sector, hospitals are primarily reimbursed based on a fee-for-service reimbursement system (Babashahy *et al*., 2016; Barouni *et al.*, 2020). Even though the Iranian PHC system has been rated as “excellent” by some international organisations, such as UNICEF and WHO (Asaei, 2014; Sajadi and Majdzadeh, 2019; Doshmangir *et al*., 2020), the PHC system needs to be revised and adjusted to emerging needs to ensure its sustainability, effectiveness, and responsiveness to public health needs (Van Lerberghe, 2008). This study aimed to explore the existing Iranian PHC system’s current challenges and examine ways forward.

## Methods

This is a descriptive qualitative study. Qualitative data were collected during face-to-face interviews with experts. A pre-defined semi-structured interview guide was developed using the WHO health system’s building blocks framework (World Health Organization, 2010) to depict services provision, workforce, health information systems, medical and technical products, financing, and leadership of the Iranian PHC system. Four pilot interviews were conducted to revise and finalise the interview guide’s content. Two experienced research team members conducted interviews. Twenty-six participants were selected using purposive and snowballing sampling approaches among policymakers, academics, managers, planners and experts of public health and PHC at the national, provincial, and international levels (respondents’ characteristics in Table 1). The main inclusion criteria for the interview was having work or research experience in PHC. There were no specific exclusion criteria. Interviews were conducted between June and September 2019, updated in February 2020, and lasted about 30–130 min. Data collection continued until saturation was reached.

**Table 1.** Demographic information of participants

Variable		*n*
Gender	Male	21
	Female	5
Age	30–40	7
	41–50	11
	51–60	8
Educational attainment	High school diploma	1
	Professional	2
	Associate	4
	Bachelor	4
	Master	4
	Doctor of medicine	4
	Doctor of Philosophy(Ph.D)	6
Work experience	5–10 years	9
	11–20 years	17
Organisation	Ministry of Health&Medical Organization	6
	University of medical sciences	4
	Insurance Organization	5
	Health network	7
	Health Center	4

All interviews were transcribed verbatim immediately after the interviews. We then used a deductive content analysis approach assisted by MAXQDA *12* software for data analyses (VERBI Software 2015, MAXQDA *12*, computer program, VERBI Software, Berlin). The interviews were coded, and the codes with close concepts were integrated. The created sub-themes were placed under the health system’s building blocks from the WHO’s framework (Table 2). We used five criteria purported by Guba and Lincoln (1994), including credibility, authenticity, transferability, confirmability, and dependability, to evaluate the credibility and trustworthiness of the study (Guba and Lincoln, 1994). To ensure data authenticity, sufficient time was allocated for data collection. At the end of each session, transcribed texts were sent to the interviewees for confirmation, and necessary revisions were made where necessary. To check the credibility, the data were given to another researcher, and the similarity of the findings of the two people was compared. To check the confirmability of the data, sampling with maximum diversity of different employees was performed. Codes prepared from the interviews were provided to the participants and were approved after the necessary amendments, if any, were implemented. To ensure transferability, we explained all the stages of the project and the study’s environment and context to the participants. To ensure the dependability of the study process, professors with experience in qualitative research reviewed the research results after reviewing the study process.

**Table 2.** The themes and sub-themes related to the Iranian PHC system

Themes	Subthemes
Leadership and Governance	Service provision structure
	Responsiveness
	Policy making and strategic planning
	Cooperation and coordination
Health financing	Collection of financial resources
	Resource pooling
	Purchasing
Medical and technology products	Health technology
	Supply of equipment
Health information	Statistical information
	Information software’s
Health workforce	Motivation
	Effective communications
	Planning and supply of human resource
	Capacity building
Health services	Health promotion
	Developing FP and referral system
	Health care rationalisation
	Provider-client relationship
	Expansion of service packages

In addition to the interviews, we used document review to enhance data triangulation. The documents included main published books in the Iranian PHC, such as the comprehensive public health book (Hatami *et al*., 2004), a new way to health, city health and treatment network (Pilehroodi, 2006), health for all and PHC in the 20th and 21st centuries (Shadpour, 2002), general terms and principles in the structure of development plans (2007), health networks in cities (1997), family physician guide (2018), the executive order of the program for providing and promoting the PHC in the urban and marginalised areas (2014).

### Ethical considerations

Written consent was obtained from all participants before starting the interviews. Participants were assured of the confidentiality of the information and informed about their right to withdraw from the study at any data collection stage. The objectives of the study were also explained to the study participants. The study was approved by Tabriz Health Services Management Research Center, Tabriz University of Medical Sciences, Tabriz, Iran (Approval No: IR.TBZMED.REC.1398.196).

### Findings

We structure our results based on six WHO health system framework’s main building blocks, including leadership and governance, health system financing, medical products and technology, health information systems, health workforce, and health service delivery (WHO, 2010).

#### Leadership and governance

Participants stressed the importance of the PHC network in health care service delivery. They stressed PHC’s unique ability to serve as a medium in providing cost-effective integrated programs through people, charities, and other intergovernmental organisations. Participants shared their dissatisfaction with the limited participation of specific key stakeholders within current Iran’s PHC system (ie, community members and inter-sectoral organisations). According to the participants, stakeholders are not involved in the design, implementation, and oversight of the PHC. Moreover, there is no specific mechanism for interesting people and cross-sectoral organizations in PHC. According to interviewees, Iran is presently experiencing a transition in disease burden, particularly in the urban setting, which necessitates a PHC approach to solving the problems. The country is facing an increasing population migration from rural to urban areas. However, the urban PHC system cannot provide new services within the current infrastructure and respond to emerging population needs. Also, parallel systems have occurred within the PHC system (health care services for armed forces, municipalities, and banks) that provide the same PHC services and create overlaps, leading to resource waste due to lack of communication.

“We have offices and health centres affiliated with various organisations such as the Armed Forces, municipalities, banks and departments that are do not have any role in the structure of primary health care but is a parallel system that does the same thing and creates an overlap. They also waste resources”. [Faculty Member]

Simultaneously, the regional managers also lack the authority to influence decision-making. Furthermore, there is limited institutional support to steward the delivery of health care services by the private sector at the local level. As a result, despite the high demand and increasing people’s need for PHC services by the private sector, the private sector does not have a good place in the structure of PHC in Iran, mainly because the current PHC services delivery is not flexible enough to adapt to required changes.

“Health challenges like cardiovascular diseases, accidents, cancer, and diabetes in urban areas are rising, but the PHC system seems to focus more on the women and children’s health in the villages (rural areas). In fact, we are not accountable”. [Health policy maker]

Moreover, interviewees asserted that significant decision-making bodies, especially health policymakers and academic institutions, focus primarily on curative and clinical care, not public health and PHC activities. A lack of coordination among public health specialists and clinicians hinders continuity of care across care levels. Interviewees also highlighted the disconnect between decision-making at the central level, which is frequently not evidence-based and execution at the local level by local managers.

#### Health financing

Interviewees asserted that the Iranian PHC system continues to experience immense financial stress. They mentioned that there is also an unfair distribution of resources in the health system. For example, most resources are allocated to treatment and related procedures. As a result, the public health or PHC network system lacks sustainable financing. Furthermore, the insurance package is not well integrated into the PHC system. As a result, PHC services are still costly, even in low-income areas. Participants also shared that this burden can be addressed when the private sector manages PHC, including outsourcing.

“Financial resources are one of the blind spots we have. The health system has no sustainable resources, and as a result, resources contribution to the primary health care is minimal”. [Provincial health centre policy maker]

“We are stingy when it comes to outsourcing. The government wants all workers, employers and even the auditors to be from the public sector, does not use the private sector’s capacity and does not have a comprehensive model for outsourcing services to the private sector. It is, therefore, obvious why we are not making any meaningful progress.” [National level Health policy maker]

#### Medical products and technology

Participants stated that one of the significant challenges facing the PHC in Iran is the excessive introduction of new sophisticated technologies without necessary training and capacity building. Most health personnel are unfamiliar and untrained to utilise the latest equipment. Some believed that advanced technologies at the PHC level led to a shift in the understanding and approach to addressing health demand at the local levels, that is, there is now a reduction in interest in promoting public health activities. Some interviewees expressed concerns that new diagnostic and therapeutic technologies and equipment are introduced without consultation with local specialists and accounting for the local context. At the same time, public health centres still lack essential health equipment.

“New technologies are now being used indiscriminately without thoroughly evaluating their effectiveness, efficiency, and safety. Besides, the public’s perceptions about prevention programs have changed – it seems everyone is looking for immediate health response (clinical remedy).” [Faculty Member]

#### Health information system

Iran has implemented several information systems aimed at facilitating better decision-making. According to participants, the country’s integrated health systems and electronic health records are still in their early implementation phases. Because of that, most PHC staff are still not familiar with how the systems work. Furthermore, participants believe that integrated health systems is not well integrated into the PHC health system and not aligned with PHC priorities and, therefore, doubt the accuracy of data produced and used to guide decision-making.

“The system [integrated health systems] demands a well-functioning Internet connection, telephone, and staff skills to achieve intended purposes which are not fully available. There is also the need to change physicians’ perception about the importance and benefits of using it.” [Expert of the University of Medical Sciences]

#### Health workforce

Participants highlighted PHC challenges related to the health workforce associated with the first level of PHC. More specifically, community health workers are typically selected from indigenous people familiar with local cultural traditions and the population’s behavioural and social characteristics in the catchment areas. However, they have poor working conditions (eg, lack of living and welfare facilities), which is the main issue preventing them from staying in the villages and leaving their jobs. According to participants, the lack of a working system for continued professional development and professional growth prospects is also demotivating. In particular, interviewees stated that human resources are unevenly distributed in the health system, especially in rural PHC facilities that cannot adequately respond to the population’s emerging health demands due to skillset and staff shortages. Increased literacy among the population and easy access to information on the Internet reduce acceptance and trust in health workers and affect interpersonal relationships. Moreover, the uncontrollable relocation of the population hinders follow-up activities and disrupts the PHC network services provision continuity. Furthermore, PHC services in Iran are perceived to be rural-driven.

On the one hand, despite the availability of health graduates in rural areas, there is no mechanism to involve them and to replace the less qualified staff. University graduates are usually not familiar with the existing problems in the rural PHC system. They lack the necessary skills that would enable them to respond to local population health needs.

“The training offered to the university graduate does not fully contribute to improved skills needed in the field.” [City health centre officer]

Although regional managers are in the rightful position to ensure even distribution of health professionals’ right mix, they lack the legal support. The inappropriate strategy of employing less trained and underqualified staff continues. Community health workers or Behvarzes training program is quite outdated and is not compatible with emerging population needs and services. In recent years, no attention has been paid to health workers’ career advancement, and their literacy level is lower than the average level of society.

“Our health workers were trained in the 1960s–1970s, and they were not trained to provide new services. So, in the current situation, they do not meet the population’s needs and cannot always adapt to modern science.” [Senior health policy maker].

Participants also stated that a fairer payment system should be established for PHC health care providers. They also recommended designing and implementing a mechanism for career advancement and motivation of health workers. Necessary human resources should be provided and trained before introducing any new service. The delegating authority to review the organisational chart should be given to local managers. Medical science education’s content and the system should be updated, catered to the population’s needs, and evidence-based. By changing the standards, Behvarzes should be recruited from university graduates and health schools should train Behvarzes.

#### Health services delivery

Participants emphasised family physicians and health promoters’ vital role in PHC health service delivery, especially in resource-deprived communities. However, interviewees also pointed to inactive engagements of several essential stakeholders, particularly the public health professionals. This negatively influenced the execution of the Health Transformation Plan, launched in 2014, particularly in controlling and unionising medical groups, causing the program to deviate from its original goals. As a result, more resources were diverted to treatment, and attention to prevention and PHC among officials and the public ceased.

“…. The absence of some actors negatively affected the implementation of the health development plan. (For example), we did not have an environmental health and health education before the development plan.” [Health policymaker]

The inappropriate design of service levelling has led to a long and complex care path. Specialised services are not required or are sometimes inclined to comply with service provision assigned to their level and provide services not typically associated with their level. According to the policymakers, physicians and patients, there is no solid structure for establishing a family physician program and a referral system. The family physician program managers are not thoroughly familiar with the program’s goals and focus more on achieving quantitative indicators. Health team members also do not seem to interact well with each other. Therapeutic physicians cannot work as family physicians and are not interested in prevention and care programs. Family physicians are typically salaried per capita, and their pay is dissociated from any other health outcomes or indicators.

“The family doctor program in our country is incomplete. Physicians graduate from the university with a “therapeutic vision” – they do not actively engage in health care and do not show interest in health promotion and prevention”. [senior health insurance officer]

While participants believe that community members can contribute enormously to health, assertions were made that the healthcare educational platforms to empower community members are inadequate. Most PHC programs implemented in urban settings have been unsuccessful. Also, male patients are not active actors or users of PHC services.

“Currently, the most common health problems and causes of death (cardiovascular diseases, accidents, cancers, diabetes, etc.) are more common in urban areas, especially among men. However, where is our system active? Mainly in the villages and for women and children. In fact, we do not have the necessary accountability ” [Health policy maker].

## Discussion

We explored the challenges faced by the Iranian PHC system using the health system framework developed by WHO. Our findings revealed that despite the brilliant history of Iran’s PHC system, there is now a considerable scrimmages with a focus on public health services to the neglect of the potentioal role of the private sector. This has contributed to low community participation in health services and lack of appropriate response to new health needs - a challenge which is heightened in times of crisis, e.g, COVID-19 pandemic.

Thus, the private sector is inadequately involved in PHC, therebey limiting its effectiveness, despite existing evidence that engaging the private sector in public PHC to create a public-private mix could strengthen PHC (Russo, 1994; Palmer, 2000; Thomas *et al*., 2016). International Finance Cooperation (2011) also recommends deliberate and systematic collaboration of the public and the private health sector in line with national health priorities (World Bank, 2011). To move toward UHC, it is necessary to harness all public and private health services while focussing on PHC (World Health Organization, 2018b). The lack of flexibility within the institutions to implement essential changes to ensure that the population’s growing health needs are met also adds to the existing challenge.

Our findings are in line with previous studies that identified similar challenges and highlighted the need for structural reforms in PHC (Donato and Segal, 2013; Khangah *et al*., 2016; Manyazewal, 2017) and ensure that PHC systems are dynamic in response to growing population needs, particularly in urban areas (Pablo *et al*., 2007; Javanparast *et al*., 2018). The inability to respond to emerging population needs in Iran gradually weakens the system. In line with our findings, other studies have reported concerns about the diminished level of monitoring of PHC functioning and the inadequacy of evidence-based initiatives (Esmailzadeh *et al.*, 2013; Malekafzali, 2014). Moreover, parallel service provision, where duplicate services to PHC are provided exclusively to employees of municipalities, banks, the ministry of oil, and the judicial system, further undermines the PHC system (Alizadeh *et al*., 2013; Damari *et al*., 2014; Heshmati and Joulaei, 2016).

The centralisation approach is dominant in the Iranian PHC system. We found that decentralisation could enable a better health system in Iran. Studies in Iran and other countries have also emphasised the importance of the decentralisation approach in service provision (Collins and Green, 1994; Nikniyaz *et al*., 2006; Alizadeh *et al.*, 2013; Damari *et al.*, 2014; Long *et al.*, 2018; Abimbola *et al.*, 2019). Our study also confirmed that PHC is currently underfinanced and underresourced. Although Iran’s PHC system is fully government-funded, there is no separate financing model for the PHC system. Such an arrangement can threaten the sustainability of the PHC system (Heshmati and Joulaei, 2016).

Overall, the PHC system seems to be one of the Iranian health system’s vulnerable areas because most of the budget is allocated for extensive training of medical professionals and providing health care and hospital-based facilities, not PHC (Malekafzali, 2014). We also found that salary of PHC professionals is not based on the level of performance and service delivery by the staff. Other studies support our findings (Nikniyaz *et al.*, 2006; Khangah *et al.*, 2016; Manyazewal, 2017) and confirm that different salary payment models in the Iranian health system could cause injustice in payments. Hence, the current payment system should be revised and improved. Some scholars also suggested that focussing more just on the salary payment in the Iran PHC system could decrease the quality and efficiency of health services (Jabari *et al.*, 2007; Heshmati and Joulaei, 2016).

While the use of technologies can foster effective health interventions (Manyazewal, 2017) and strengthen the health systems (Khangah *et al.*, 2016), we found limited evidence about innovations aimed at improving PHC in Iran. Studies have highlighted the ever-increasing need and urgency to scale up the implementation of piloted digital health interventions (Stevanović *et al.*, 2005; Long *et al.*, 2018; World Health Organization, 2018a) and the provision of an appropriate platform for consistent capacity building of health professions on the use of health technologies (Ayatollahi *et al.*, 2015; Hone *et al*.). Our results also renew the relevance of assessing the effectiveness of these health technologies to ensure that products or services effectively meet the population’s health needs. Poor health information systems are another technological challenge of the Iranian health system. Our findings showed a lack of data coherence which threatens the reliability of information used in decision making. In line with information technology, the statistical system has not evolved, and outdated tools for recording, analysing and reporting data are still being used. Moreover, most health professionals lack electronic data management skills (Asadi *et al.*, 2012; Moghaddam *et al*., 2013; Mehrolhassani *et al*., 2018).

In Iran, most PHC workers are expected to be familiar with the community’s norms and culture in which they work. As a result, community health professionals are recruited from the community they live. This approach contributed enormously to the positive interpersonal relationship between health professionals and clients at the PHC level (Aghajanian *et al.*, 2007; Javanparast *et al*., 2011a). In Cambodia, China, and Vietnam, socio-economic development provided personal and professional support to attract and retain rural health workers (Zhu *et al.*, 2019). Moreover, it was shown that to improve the health status of rural Australians, specific rural health workforce skills are required, together with a sound understanding of the rural context in which they are operating (ie, rural culture and ethos, attitudes, behaviour, and lifestyle behaviour) (Humphreys *et al*., 1998).

Nonetheless, in Iran, communities experience challenges in having access to the right mix and dynamics of health professionals needed because of the uneven distribution of human resources. Furthermore, the discretion of organising staff and shifting the organisational chart does not fall within the scope of local and regional health directors. Academic medical education is not responsive to the current population’s health needs and expectations, particularly at the PHC level. Graduate students have inadequate knowledge and skills needed to perform their duties. For example, about 54% of family physicians reported a difference between students’ education and expectations of a family physician (Karimi *et al.*, 2012). As a result, university education was previously recommended to contain PHC-based training (Nekoei Moghadam *et al*., 2018). Unfortunately, the curricula are also not being revised consistently to ensure that health professionals have advanced training to meet identified health needs and changing expectations of the populations (Sharifi-Yazdi *et al.*, 2015). Hence, recruitments procedures should be revised to attract university graduates to work at the PHC levels to improve health outcomes.

Although the family physicians program is a significant component of the Iranian PHC system (Mehryar, 2004; Takian *et al.*, 2013; Zanganeh Baygi *et al*., 2016), the initiative has not received the needed attention. The physicians’ therapeutic outlook and inability to work as family physicians (or general practitioners) led to a reluctance to perform health care. Again, research findings indicated low community participation in health care delivery. Besides, the government is responsible for taking major health decisions (Sheikhattari and Kamangar, 2010; Acosta Ramírez *et al.*, 2016), and not the local and regional health directors. Other studies also indicated that providing services via PHC in urban areas is inactive and frequently unsuccessful (Lionis and Philalithis, 2008). It has also been confirmed that PHC in urban areas faces problems such as a lack of public visits, population knowledge, and sufficient information about health and population morbidity.

Our study has implications for health policymakers and planners, which addressed the weaknesses and challenges of the existing PHC system. Our study implies that the functions of the PHC system need to be revised to address the challenges of the current system. This study has some strengths and limitations. Our study involved participants with considerable knowledge and experience of the past and present status of the Iranian PHC system. The WHO framework enabled us to accommodate a wide range of disciplines for pragmatic interpretation of the PHC in Iran. However, our major limitation is that we could not be sure of the interviewees’ primary rationale behind their responses and their information. For example, their views can be biased and reflect their existing institutional commitments or the political and societal role throughout the policymaking cycle. But, we tried out best to interpret the findings carefully and cautiously.

## Conclusion

Community health needs and expectations are changing rapidly. This requires an effective PHC network capable of responding to health demands comprehensively. Well-trained family physicians, active community participation, private sector engagements, and active involvement of inter-sectoral and non-governmental organizations are fundamental to a well-function PHC network in Iran, especially in delivering evidence-based initiatives. But to foster sustainable progress, we recommend: the design of a comprehensive model for outsourcing transferable PHC services; an accreditation model for units and centres providing PHC services; and the provision of a periodic evaluation model for adapting the structure and services of the PHC system to the needs of the community.
